# Autoantibodies Against C3b—Functional Consequences and Disease Relevance

**DOI:** 10.3389/fimmu.2019.00064

**Published:** 2019-01-29

**Authors:** Vasil V. Vasilev, Maria Radanova, Valentin J. Lazarov, Marie-Agnes Dragon-Durey, Veronique Fremeaux-Bacchi, Lubka T. Roumenina

**Affiliations:** ^1^Nephrology Clinic, University Hospital “Tsaritsa Yoanna-ISUL,” Medical University–Sofia, Sofia, Bulgaria; ^2^Department of Biochemistry, Molecular Medicine and Nutrigenomics, Medical University Varna, Varna, Bulgaria; ^3^Assistance Publique–Hôpitaux de Paris, Service d'Immunologie Biologique, Hôpital Européen Georges Pompidou, Paris, France; ^4^INSERM, UMR_S 1138, Centre de Recherche des Cordeliers, Paris, France; ^5^Sorbonne Universités, UPMC Univ Paris 06, Paris, France; ^6^Université Paris Descartes, Sorbonne Paris Cité, Paris, France

**Keywords:** anti-C3 autoantibodies, anti-C3b autoantibodies, complement, immunoconglutinins, autoimmunity, systemic lupus erythematosus, lupus nephritis, C3 glomerulopathy

## Abstract

The complement component C3 is at the heart of the complement cascade. It is a complex protein, which generates different functional activated fragments (C3a, C3b, iC3b, C3c, C3d). C3b is a constituent of the alternative pathway C3 convertase (C3bBb), binds multiple regulators, and receptors, affecting thus the functioning of the immune system. The activated forms of C3 are a target for autoantibodies. This review focuses on the discovery, disease relevance, and functional consequences of the anti-C3b autoantibodies. They were discovered about 70 years ago and named immunoconglutinins. They were found after infections and considered convalescent factors. At the end of the twentieth century IgG against C3b were found in systemic lupus erythematosus and recently in lupus nephritis, correlating with the disease severity and flare. Cases of C3 glomerulopathy and immune complex glomerulonephritis were also reported. These antibodies recognize epitopes, shared between C3(H2O)/C3b/iC3b/C3c and have overt functional activity. They correlate with low plasmatic C3 levels in patients. *In vitro*, they increase the activity of the alternative pathway C3 convertase, without being C3 nephritic factors. They perturb the binding of the negative regulators Complement Receptor 1 and Factor H. The clear functional consequences and association with disease severity warrant further studies to establish the link between the anti-C3b autoantibodies and tissue injury. Comparative studies with such antibodies, found in patients with infections, may help to uncover their origin and epitopes specificity. Patients with complement overactivation due to presence of anti-C3b antibodies may benefit from therapeutic targeting of C3.

## Introduction

The complement system is apart of the innate immune defense (1, 2). Autoantibodies against complement components and regulators have proven pathogenic effect. These antibodies (Ab) may cause acquired functional deficiencies of the complement cascade or induce amplification of an already activated complement (3–6). This review is focused on the auto-antibodies, recognizing the activation products of the central complement component C3. The history of their discovery, their prevalence in different diseases as well as their functional and clinical relevance are discussed. Multiple unanswered questions open avenues for future studies.

## The Complement Component C3—Structure and Function

**Figure 1:**
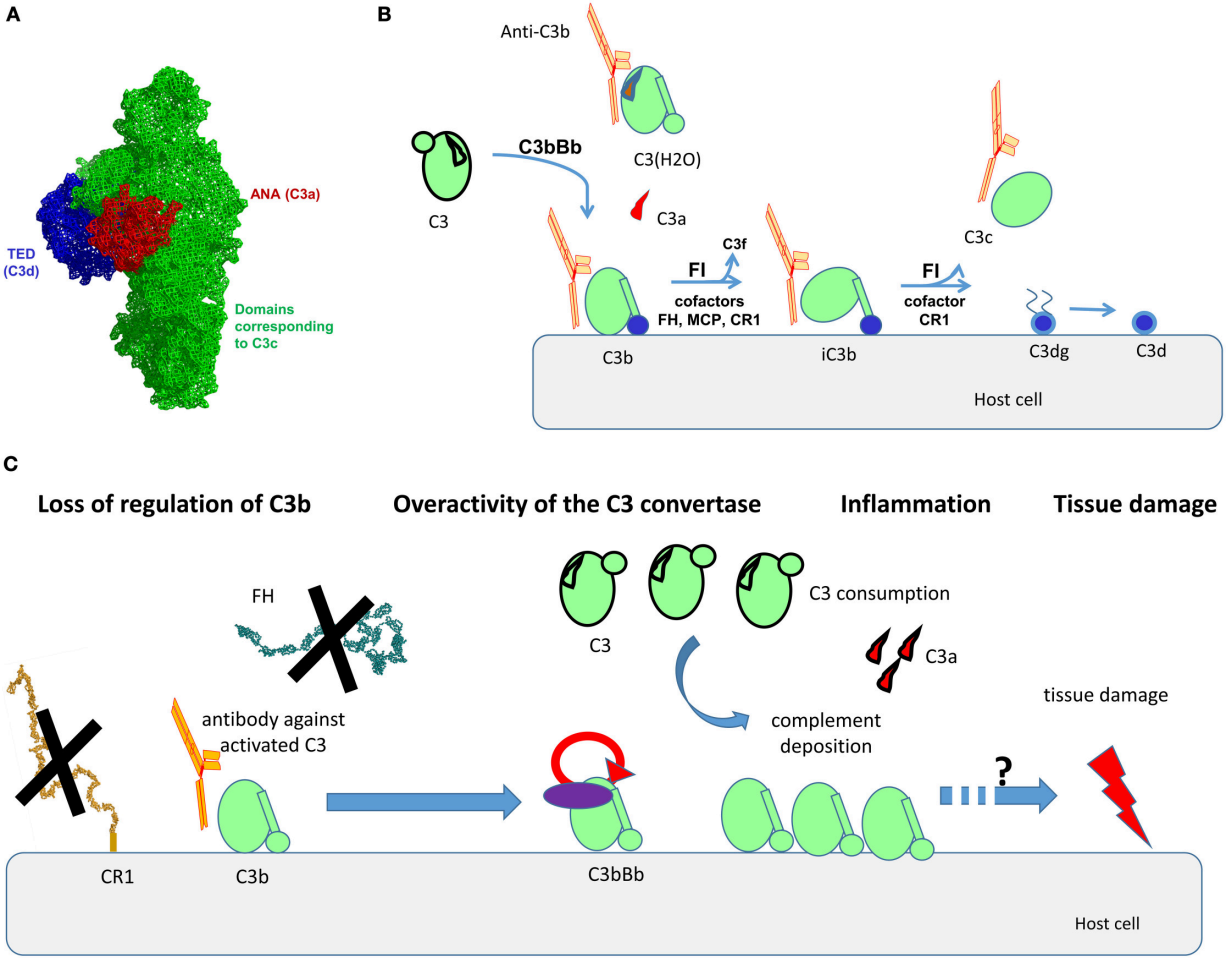
Structural organization of C3, its activation forms, localization of the binding epitopes, and proposed mode of action of the anti-C3b auto-antibodies. **(A)** Structural organization of C3, based on the crystal structure published in Janssen et al. (8). The different domains, corresponding to the different fragments of the molecule are depicted with different color. Of note are the ANA domain, corresponding to C3a (red), the domains corresponding to C3c (green), and the TED domain, corresponding to C3d (blue). **(B)** Steps of cleavage of C3 by the C3 convertase of the alternative pathway C3bBb and generation of its activation forms. The cofactors needed for the cleavage of C3b by Factor I (FI) are indicated. The activation forms of C3, recognized by the autoantibodies (C3(H2O), C3b, iC3b, C3c) are indicated as complexed with an antibody in orange. **(C)** Proposed mode of action of the anti-C3b autoantibodies. The Ab will bind to C3b, preventing its interaction with CR1, and in the context of lupus nephritis, with Factor H (FH). This loss of regulation, together with the direct stabilization/enhancement of formation of the alternative pathway C3 convertase C3bBb will result in overactivation of complement, generating inflammation (via C3a), massive deposits of C3 activation fragments and finally, tissue damage. The exact mechanism linking complement overactivation to tissue injury and its contribution to organ injury need further studies.

C3 is the central component of the complement system (7). It is the convergent point of the three pathways of the cascade (1, 2). C3 is a 185-kDa glycoprotein, which belongs to the α2-macroglobulin family, part of the thioester-containing protein superfamily. C3 consists of two polypeptide chains—α-chain (110 kDa) and β-chain (75 kDa), linked by one disulfide bond and by non-covalent forces (8). It contains a globular thioester domain (TED) with an intrachain thioester bond, capable to attach covalently to surfaces; eight macroglobulin (MG) domains, and CUB domain, which frames and holds the TED domain (Figure 1A).

C3 undergoes low-rate spontaneous conformational change, leading to hydrolysis of its thioester bond (9). This generates an activated form of the protein, called C3(H2O). It does not bind to surfaces (as it no longer possesses a thioester group), but it resembles C3b in many of its functional and structural features, despite its retention of the ANA domain. C3(H2O) is the initiator of the alternative pathway, since it binds Factor B (FB) and Factor D (FD) and generates a fluid phase initiating convertase C3(H2O)Bb, capable to cleave C3. C3 is cleaved also by the classical or alternative pathway surface C3 convertases to generate C3a (9 kDa), a small potent pro-inflammatory molecule, and C3b (177 kDa) (Figure 1B). Generated C3b changes conformation, uncovering the binding sites for its ligands, including FB, Factor H (FH), complement receptor 1 (CR1, CD35), properdin, etc. (10). C3b interacts with Factor B to form the C3 convertase of the alternative pathway C3bBb (11, 12). Properdin is the only positive regulator of complement, stabilizing the convertase (13). The activity of C3b is tightly regulated to avoid accidental host tissue damage by negative complement regulators, like membrane-bound CR1, membrane cofactor protein (MCP; CD46), decay acceleration factor (DAF; CD55) or circulating regulators like FH, Factor-H-like protein 1 (FHL-1), and Factor I (FI) (14, 15). FI cleaves C3b in presence of cofactors (like the regulators MCP, FH, CR1) to generate iC3b (inactivated C3b, unable to form convertases). In presence of CR1 the cleavage by FI proceeds to C3dg and finally C3d fragments, remaining attached to the surface. When iC3b is cleaved, C3c is released (Figure 1B).

C3 activation fragments not only participate in cleaning of pathogens, apoptotic cells, and cellular debris but also in a number of homeostatic processes. This includes tissue regeneration, synapse pruning, controlling tumor cell progression, etc. (7). Therefore, alteration of the function of the activation fragments of C3 by genetic abnormalities results in pathological conditions (7). Indeed, mutations in the *C3* gene result in an abnormal protein, promoting complement overactivation and predisposing to renal injury (atypical hemolytic uremic syndrome) due to loss of regulation or direct overactivation of the C3 convertase (16–18). On the other hand, complete C3 deficiency shows increased susceptibility to bacterial infections in early childhood (19). Critical role of intracellular C3 activation for T cells function was recently described (20). This intracellular C3 activation, as well as the C3-based recycling pathway and C3 being a driver and programmer of cell metabolism suggest that the complement system utilizes C3 to guard not only extracellular but also the intracellular environment (21).

The activation products of C3 are also a target for autoantibodies. The following parts if this review describe the functional consequences and clinical relevance of the autoantibodies targeting C3b.

## From Immunoconglutinins to Anti-C3b Ab

The notion of Ab, recognizing activated forms of C3, dates from the mid-twentieth century, when they were named immunoconglutinins (22). Like rheumatoid factors are Ab binding to IgG, immunoconglutinins are Ab, binding components of complement. By definition, the immunoconglutinins are a group of Ab, formed in response to antigenic stimulation by components of an animal's own fixed complement components C3, but sometimes C4. They react against newly-formed epitopes, created after activation of C3 and C4, when the proteins change their conformations. Immunoconglutinins appear after viral or bacterial infections, the titers peak about 2 weeks following infection and usually drop rapidly afterwards (23–26). The Ab are frequently from IgM class. In chronic infections in animal models, high titres immunoconglutinins persisted over a prolonged period of time (27). At this period, it was concluded that the immunoconglutinins are convalescence factor, helping the healing process (25). Indeed, pretreatment of mice with immunoconglutinins prior to challenge with virulent strains of bacteria resulted in prolonged survival and decreased mortality (28). It was hypothesized that immunoconglutinins could enhance the clearance of bacteria by phagocytes.

This view was challenged, when immunoconglutinins/anti-C3 activated forms Ab were established in patients with autoimmune diseases. Such Ab were detected in systemic lupus erythematosus (SLE) (29–32), lupus nephritis (LN) (33, 34), in Crohn disease (35), in some nephrotic kidney diseases (36–38), in dense deposit disease (DDD) (39), in C3 glomerulopathy (C3G), and Immune Complex glomerulonephritis (IC-GN) (40) as well as in autoimmune-prone mice (32). However, these Ab has not been detected in primary biliary cirrhosis or rheumatoid arthritis (30, 32). A single patient with atypical hemolytic uremic syndrome, positive for anti-C3b Ab was also reported (41). These Ab were IgG and were measured as anti-C3 or anti-C3b Ab by ELISA (29, 31–34, 39–41) or as immunoconglutinins (36–38). In SLE they are predominantly belonging to IgG1 and IgG3 subclasses (30).

## Clinical Relevance of the Anti-C3b Ab

### SLE and LN

Systemic lupus erythematosus is a heterogeneous, multisystem autoimmune disease (42). Kidney involvement in SLE, also known as LN, is a common and serious organ complication that determines the quality of life and prognosis in patients with SLE and is characterized by specific clinical (nephritic or nephrotic syndrome), laboratory (proteinuria, hematuria), immune, and morphological (proliferative or non-proliferative glomerulopathy with mesangial, subendothelial, and subepithelial deposition of immune complexes, tubulointerstitial, and vascular lesions) manifestations. Different disorders of the regulation of the immune response with production of a wide range of Ab directed to various self-antigens (DNA, nuclear proteins, ribosomal proteins, and complement component C1q), are among the main characteristics of SLE and LN. The complement system plays a critical role in inflammatory and immune responses, in clearance of immune complexes and apoptotic cells, and autoreactivity to complement may have considerable pathological consequences (1, 2). The classical pathway has a predominant role in the initiation of the complement activation in SLE and LN, but the complement-mediated damage is often caused by the alternative pathway amplification loop (43).

Most of the reports in the literature related to anti-C3b concern cohorts of SLE or LN. The levels of anti-C3b Ab (measured as immunoconglutinins) were higher in active SLE compared to patients with inactive disease (29). We reported for the first time that more than 30% of LN patients (12/39) were positive for anti-C3 Ab (measured against C3 immobilized on an ELISA plate and confirmed against C3b) (33). Here again the levels of anti-C3b Ab were higher in the LN patients with severe disease, compared to a milder one. In the anti-C3b Ab-positive LN patients, the plasma levels of C4 and C3 were also lower compared to the negative ones, showing thus association of these Ab with complement activation. An additional case of LN revealed positivity against C3b, but also against FI, FB, C3, and properdin (44). The patient carried also a heterozygous mutation in the gene of C3.

In the same time Birmingham et al. showed in a bigger cohort an association of Ab against C3b with LN activity and their diagnostic and prognostic value (34). In this study, among 74 LN patients, 36% were positive for anti-C3b Ab, while only 1 out of 41 cases (2%) with non-renal SLE showed such positivity. Here again, the presence of anti-C3b Ab correlated with plasmatic C3 consumption. In the cross-sectional assessment, compared with anti-C1q IgG, anti-C3b IgG was less sensitive but more specific for lupus nephritis. In a longitudinal analysis, the rise of the levels of anti-C1q Ab were prognostic markers for LN flare only in anti-C3b Ab positive patients, thus defining anti-C3b Ab as a useful marker for to identify at risk patients for disease flare (34).

The presence of ant-C3b Ab correlated with the presence of anti-dsDNA (33). It was hypothesized that the dual presence of anti-dsDNA and anti-C1q Ab may coincide with, and possibly drive, a level of complement activation that leads to the onset of anti-C3b Ab (45).

### C3G and IC-GN

C3G is a renal disorder characterized by the presence of glomerular C3 staining in the absence of significant immunoglobulins staining (46). Subsets of C3G include DDD and C3 glomerulonephritis (C3GN). C3G is marked by the presence of glomerular deposits. They are electron dense (by electron microscopy) and localized in the lamina densa of the glomerular basement membrane in case of DDD or are subepithelial and subendothelial in patients with C3GN. The deposits in C3G contain complement components of the alternative pathway—C3b, iC3b, C3dg, C3c (47). The dysregulation of the alternative complement pathway, often caused by the presence of C3NeF, maintains a permanent activation of the complement (48).

In a cohort of 141 patients with C3G and IC-GN, only eight patients were positive for anti-C3b Ab, among which 5 were also positive for anti-FB Ab (40). These eight patients showed increased Bb fragment in plasma. Interestingly, the patients positive for anti-C3b Ab and anti-FB Ab in this cohort had higher rates of infections. Another study described two DDD patients with combined anti-C3b and anti-FB Ab (39). There is still not sufficient data that could determine the diagnostic value of anti-C3b Ab in patient with C3G and IC-GN.

## Methods of Detection and Binding Epitopes

Nowadays the presence of anti-C3 activation fragments Ab can be detected routinely by ELISA (31–34, 40). More in depth characterization of the binding is done by surface plasmon resonance (SPR), allowing evaluation of the antigen-antibody interaction in real time as well as running functional tests for the formation, and regulation of the C3 convertase (33, 40, 49).

From the early studies it was known that the immunoconglutinins do not recognize native C3, but rather interact with its activated forms (22). Later studies revealed binding to immobilized C3 (hence C3(H2O)-like); C3b, iC3b, C3c with variable intensity but rarely to C3d and C3a (30, 33, 40). Therefore, the screening for diagnostic purpose should be done using coating with C3b, since it contains all recognized epitopes. C3 was also used as an antigen for the ELISA of detection (33), since it changes its conformation upon immobilization to the plastic, adopting most likely C3b(H2O)-like appearance, revealing the binding epitopes (8, 10). In some patients, positive for anti-C3b Ab, reactivity against immobilized C4 was detected as well (31, 33), but it is difficult to conclude whether these are separate Ab or a cross-reactivity of the anti-C3b ones. Nevertheless, all LN patients, positive for anti-C3b were negative for anti-C4b Ab in one of the published cohorts (34), suggesting that anti-C4/C4b Ab are most likely a separate entity occurring in some patients.

The anti-C3b Ab are different than the C3 Nephritic factor (C3Nef), since by definition C3Nef binds to the convertase C3bBb but not to its isolated components C3b and Bb (50). For one patient with very high titers of anti-C3b Ab, the C3Nef functional test remained negative (33). Moreover, anti-C3b Ab, together with Ab against FB, were detected in two unrelated patients with DDD without C3NeF activity (39).

The complexity of the C3 structure and the dramatic conformational change that the protein undergoes during its cleavage steps expose neoepitopes, which may drive pathological immune response. The ensemble of the results for the epitope mapping suggests that the immunodominant epitope is located within the domains, corresponding to the portion of the protein, which will become C3c after cleavage. This is supported by the fact that in the majority of the cases the reactivity is revealed toward C3b, immobilized C3, and C3c but rarely C3a and C3d (30, 33, 39–41). The domains corresponding to the C3c portion within C3b represent the central part of the protein, where are located the binding sites for FB (12) and numerous negative regulators, as FH, CR1, MCP, and DAF (14), Figure 1B. These data are relevant only to the anti-C3b Ab, found in autoimmune disease. Unfortunately, there is no epitope data about these Ab, occurring after infection. It is still unclear how the anti-C3b Ab originate and what is the immunization mechanism, but it could be explained by immune response to C3b neoepitopes. It was found that C3 could be phosphorylated at various sites, which influence its functional activity (51). It could be a reason for immunogenic properties of molecule, because increased levels of phosphorylated C3 are detected in SLE patients (52).

The activation products of C3 have diverse binding partners and functions, therefore it may not be surprising that acting on one epitope, the antibody will facilitate the pathogen clearance and on another—the complement overactivation, loss of regulation, and tissue damage. Another hypothesis could be that the binding epitopes are similar in both cases. In physiology, complement-overactivating anti-C3b IgM may help to clear the infection and disappear afterwards. Nevertheless, if they persist over time, switch to IgG, undergo epitope spreading, and reach high titers, the anti-C3b Ab could be of harm. This will be particularly relevant in autoimmune-prone background. Alternatively, C3 activation within the kidney may result in a C3b deposited in a way that presents neoeptiopes that drive anti-C3b IgG production.

## Functional Consequences

It is critical to determine whether the anti-C3b Ab are protective, a disease-relevant factor or a simple epiphenomenon, one among many, arising from the dysregulated immune response in the autoimmune diseases (53, 54). Although limited, the experimental studies clearly describe functional consequences for these Ab (Table 1). It was found that anti-C3b positive IgG, purified from plasma of patients with LN, SLE or C3G, and IC-GN, trigger overactivation of the complement cascade by the alternative pathway (30, 33, 39, 40). The mechanism of the complement activation, however, vary depending on the particular disease. In LN, C3G, and IC-GN, IgG from the patients enhanced C3 cleavage and the formation of new convertases. Moreover, they induced C3 activation fragments deposition on the surface of endothelial cells (33, 40), which could contribute to the disease process (55). These Ab, as well as the ones from SLE patients, inhibited also the interaction of C3b with its negative regulator CR1 and the Factor I-mediated cleavage (30, 33, 40). For two SLE samples, the anti-C3b Ab also perturbed the factor I-mediated release of immune complexes from CR1 (30). Nevertheless, only the tested Ab from LN perturbed the interaction of C3b with factor H (33, 40). Interestingly, these Ab differed also in terms of the stability of the formed complexes with C3b. The ones from C3G/IC-GN showed a rapid association but fast dissociation, while the C3-Ab from LN had a slow association rate but formed more stable complexes (49). This may explain in part the apparent functional differences. Based on the available functional data, the anti-C3b Ab perturb the binding of the negative regulators FH and CR1 to C3b, causing mostly loss of regulation and to a milder degree—direct overactivation of the C3 convertase.

**Table 1:** Prevalence and functional consequences of the anti-C3b Ab in autoimmune diseases (cohort studies).

	**SLE (total)**	**SLE (non-renal)**	**LN**	**C3G/IC-GN**	**References**
**PREVALENCE OF THE ANTI-C3b**					
Cohort 1, association with LN activity, frequently associated with anti-C1q Ab	27/114 (24%)	1/41 (2%)	26/73 (36%)		(34)
Cohort 2, association with LN activity			12/39 (31%)		(33)
Cohort 3	13/53 (25%)				(32)
Cohort 4, association with activity	17/20 (85%)				(31)
Cohort 3, association with activity	11/34 (32%)				(29)
Cohort 4, frequently associated with infections and presence of anti-FB Ab				8/141 (6%)	(40)
recognized C3 fragments					(33) for LN (31) for total SLE (40) for C3G/IC-GN
C3(H2O)	17/20		4/4	8/8	
C3b	10/20		4/4	8/8	
C3c	18/20		4/4	4/8	
iC3b	15/20		4/4	n/a	
C3dg	7/20		n/a	n/a	
C3d	0/20		0/4	1/8	
C3a	n/a		0/4	1/8	
C4/C4b	n/a		3/4	3/8	
**FUNCTIONAL CONSEQUENCES**					
Inhibition of CR1 binding by SPR	n/a		7/7	4/4	(33) for LN (30) for total SLE (40) for C3G/IC-GN
Inhibition of immune complexes release from CR1	2/3		n/a	n/a	
Inhibition of FH binding by SPR	n/a		4/4	0/6	
Inhibition of FH cofactor activity	3/3		1/1	n/a	
Inhibition of the C3 convertase dissociation by FH, western blot	n/a		1/1	n/a	
C3 fragments deposition on resting endothelial cells, FACS	n/a		6/9	2/2	
Ba generation after addition to serum, ELISA	n/a		5/5	n/a	
Enhance C3/C5 convertase hemolytic test	n/a		n/a	2/7	
Enhance C3 convertase by SPR	n/a		2/2	2/2	
Inhibit C5 convertase hemolytic test	2/3		n/a	n/a	

Although the LN is clearly disease of overactivation of the classical pathway, it is important to note that the alternative pathway amplification loop is the main source of the terminal pathway complex C5b-9 (43). It is the one that generates a large number of C3b molecules that make both classical and alternative pathway C5 convertases. The capacity of anti-C3b Ab to perturb the regulation at the level of C3b would, therefore, result in an acceleration in the deposition of the C3 activation fragments, and might also result in enhanced C5b-9 deposits. The putative mechanism of action of the anti-C3b Ab is presented at Figure 1C.

Limited data is available for the action of anti-C3b Ab at the level of the C5 convertase. This process is not studied in LN. Nevertheless, anti-C3b/C3c Ab from two SLE patients inhibited the alternative pathway C3 convertase in an *in vitro* model, deprived of action of the complement regulators (30). On the contrary, in a relatively similar model, 2 out of 3 tested anti-C3b Ab from C3G/IC-GN patients enhanced C3/C5 convertase formation (40). Such data is not available for LN. Further studies are needed to elucidate the action of the anti-C3b Ab on the C5 convertase in different disease. It is tempting to speculate that in different pathological contexts the anti-C3b Ab will affect differently the convertases. In C3G, C3Nef, and C5Nef are described, acting differentially on the two convertases and having distinct disease associations (56).

Another aspect, which remains unexplored is the capacity of the anti-C3b Ab to bind to the C3b deposits in the kidney and to amplify the complement activation. This is possible in LN and IC-GN, where renal immunoglobulin deposits are clearly present and may, in part, be related to immune complexes, containing C3b as an antigen. In C3G, by definition, the patients do not have or have very limited, IgG deposited in the kidney. It is interesting to note that in C3G the presence of anti-C3b Ab is in fact, extremely rare, contrary to the high frequency in LN.

Apart from the action of the anti-C3b Ab at the level of the complement cascade, additional functions were described for these Ab. Suppression of apoptotic cell disposal by Ab against deposited C3 may contribute to increasing severity and/or exacerbations in different autoimmune diseases (32). Indeed, anti-C3b Ab blocked the recognition of C3b-opsinized cells by macrophages in a mouse model.

Further functional studies are needed in larger cohorts to determine to what extend the anti-C3b Ab have a pathogenic potential and whether they can transfer the disease, for example in a mouse model. Comparative studies are lacking for the anti-C3b Ab from patients/mice recovering from infections and the ones with autoimmune diseases. It is tempting to speculate that the functional consequences in the two contexts will be clearly different.

## Conclusion

Growing body of experimental data revealed that the anti-C3b Ab have overt functional consequences (30, 32, 33, 40) and correlate with disease severity at least in LN (33, 34). Additional clinical and experimental studies are needed to confirm their role in the disease pathogenesis and their relevance as a biomarker for the clinical practice. Nevertheless, the current state of the art shows arguments that identify the anti-C3b Ab as a potential diagnostic and prognostic marker in LN patients. The presence of anti-C3b Ab indicates a level of complement activation sufficient to initiate and accelerate the kidney damage. Therefore, these Ab should be taken into consideration in the management of LN. We hypothesize that patients with complement overactivation due to presence of anti-C3b Ab may benefit from therapeutic targeting of C3.

## Author Contributions

LR, VV, and MR reviewed the literature and wrote the first draft. VF-B, M-AD-D, and VL revised the manuscript. All authors validated the submission.

### Conflict of Interest Statement

The authors declare that the research was conducted in the absence of any commercial or financial relationships that could be construed as a potential conflict of interest.
